# Editorial: Cellular micro-environment of the endometrium: reproduction, embryo implantation, and placentation - from bench to bedside and beyond to tissue engineering

**DOI:** 10.3389/fcell.2026.1788590

**Published:** 2026-01-28

**Authors:** Ivan Varga, Victor Carriel

**Affiliations:** 1 Institute of Histology and Embryology, Faculty of Medicine, Comenius University, Bratislava, Slovakia; 2 Institute of Anatomy and Anthropology, Faculty of Medicine, Riga Stradins University, Riga, Latvia; 3 Department of Histology, Tissue Engineering Group, Faculty of Medicine, University of Granada, Granada, Spain; 4 Instituto de Investigación Biosanitaria ibs. GRANADA, Granada, Spain

**Keywords:** endometrium, human reproduction, implantation, infertility, recurrent implantation failure, uterine NK cells

It has been reported that the birth rate in the United States experienced a sharp decline of approximately 22% between 2007 and 2022. One of the contributing factors to this trend is a decrease in the general fertility rate among women. Infertility represents a global health issue, affecting approximately 15% of couples worldwide and resulting in substantial medical, emotional, and socioeconomic consequences. On a global scale, declining fertility rates combined with an increasing maternal age at first childbirth further exacerbate these challenges (Adashi et al., 2025; Szeliga et al., 2025).

There are numerous factors affecting fertility, including male-related factors such as testicular dysfunction disorders, alterations in semen production and quality (Sanci et al., 2025; Kocamaz et al., 2025), and erectile dysfunction (Yuce et al., 2026), as well as female-related factors, including ovarian and ovulatory dysfunctions (Balen et al., 2024; Saleem Azam et al., 2025), tubal factor infertility (Csöbönyeiová et al., 2022; Culenova and Kleinova, 2025), uterine adhesions from things such as Asherman’s syndrome (Eroğlu et al., 2025; Erol et al., 2025), lifestyle-related influences (Barraza-Ortega et al., 2025), or environmental exposures (Değermenci et al., 2025; Zahumensky et al., 2024). In addition, infertility is frequently associated with psychological distress, including depressive and anxiety symptoms (Chaurasiya et al., 2025; Çetintulum Aydın et al., 2025). At this point, it is also important to address and dispel widespread conspiracy theories attributing declining fertility rates to mass vaccination against SARS-CoV-2. Accumulating evidence clearly demonstrates that SARS-CoV-2 vaccination has no negative impact on fertility (Jasilioniene et al., 2025; Karakaş et al., 2025).

Based on the importance of human reproductive medicine in our society, Frontiers in Cell and Development published this Research Topic entitled “Cellular Micro-Environment of the Endometrium: Reproduction, Embryo Implantation, and Placentation - From Bench to Bedside and Beyond to Tissue Engineering”. In this Research Topic, composed of 11 articles as well as the present editorial, we focus on elucidating the “black box” of human reproduction: the embryo-endometrium crosstalk/dialogue (Kleinová et al.). Furthermore, contributors discuss the clinical impact of progesterone levels on human chorionic gonadotropin (Han et al.) and mouse decidualization (Luo et al.). Moreover, the role of the circadian clock in female embryo implantation is reviewed, hypothesizing a promising role of melatonin in the treatment for implantation failure (Zhou et al.). On the other hand, the effect of diabetes mellitus on the endometrial and uterine tube microenvironment is also discussed, highlighting glycemic control and lifestyle adaptations, as well as the potential role of stem cells, engineered models, and artificial intelligent approaches (Jackuliak et al.). Interestingly, researchers employ a multidisciplinary approach to show evidence indicative of the cytotoxic effect of polystyrene nanoplastics—as environmental exposure—on cultured trophoblast cells (Ragusa et al.), the molecular regulation of endometrial surface epithelium and gland development and function (Yang et al.), and the mediating role of extracellular vesicles in embryo-maternal cell dialogue (Mousavi et al.).

In this line, the interaction between the embryo and the endometrium can be described as a complex cellular dialogue characterized by a fine balance between immunological tolerance and immune protection, highlighting an apparent immunological paradox that serves as a gateway to successful implantation, endometrial decidualization, placental formation, and subsequent embryonic and fetal development. The embryo-endometrium crosstalk involves careful balance, and pregnancy success fully depends on successful implantation. The endometrium should be in a receptive and mature state and the competent blastocyst in a synchronized manner. Embryo-endometrium crosstalk involves adhesion, signaling, regulatory molecules (Akgün et al., 2025; Voros et al., 2025), extracellular vesicles (Kováčová Ilijew et al., 2026; Merino-Pérez et al., 2025), and immune and stromal cells (Lapides et al., 2025; Yuan et al., 2025) and can be adversely affected by a variety of factors. At the cellular level, these include, for example, reduced antioxidant capacity of embryonic or maternal cells during implantation (Kreheľová et al., 2024; Kreheľová et al., 2025) or an imbalance between cell proliferation and apoptosis during implantation (Ma et al., 2022; Golal et al., 2025). Dysregulation in this molecular and cellular dialogue can lead to implantation failure and/or complications of pregnancy (Makrigiannakis et al., 2025).

Embryo implantation is a critical step in early prenatal development to establish pregnancy and placenta formation and support embryonic and fetal growth until birth. Implantation mechanisms consist of fine-coordinated molecular processes during which the endometrial fibroblasts undergo decidualization. This is characterized by the transformation of these endometrial stromal cells into decidual cells, regulated by diverse hormones and facilitated by local immune cells (mostly uterine NK cells). Both immune and stromal cells play an indisputable role in accepting hemi-allogenic embryos. Immune cells may reach 30%–40% of the total endometrial cell population during early pregnancy and approximately 20%–30% of women with recurrent implantation failure, habitual abortions, and preeclampsia show elevated uterine NK cell count, suggesting its importance (Baharaghdam et al., 2025; Lapides et al., 2023). The endometrium as an immunological gateway to pregnancy and cellular events during implantation remains an enigma for scientists because of the difficulty involved in direct research, as the events occur inside the uterine cavity.

Treatment of patients with recurrent implantation failure, habitual abortion, and idiopathic infertility remains a challenge in reproductive medicine. The best treatment option is probably an approach based on personalized medicine with standardized endometrial immunologically active and stromal cells, with testing and targeted therapy recommendations. Although there is currently no certain indication of immune therapy’s success, it is undeniable that new, speculative, yet innovative therapeutic approaches are worth exploring, such as various intrauterine infusion treatments including intrauterine platelet-rich plasma or mesenchymal stem cells application (Cai et al., 2025; Jiang et al., 2025; Saberi et al., 2025; Nakagawa et al., 2025).

Understanding the immunological roles of diverse endometrial cell populations in reproduction is essential to comprehending the cellular mechanisms of embryo implantation. Therefore, exploring these processes and their translation into clinical practice could substantially impact the further development of the field of reproductive medicine (improvement of fertility and pregnancy outcomes) and the management of sexually transmitted infections and gynecological malignancies. Our effort through the present Research Topic of eleven articles is to increase the understanding of maternal-embryo interactions initiated at implantation and to develop clinical interventions to address the high incidence of implantation failures and miscarriages.
